# Evaluating ChatGPT's Ability to Solve Higher-Order Questions on the Competency-Based Medical Education Curriculum in Medical Biochemistry

**DOI:** 10.7759/cureus.37023

**Published:** 2023-04-02

**Authors:** Arindam Ghosh, Aritri Bir

**Affiliations:** 1 Biochemistry, Indian Institute of Technology Kharagpur, Dr. B.C. Roy Multi-Speciality Medical Research Centre, Kharagpur, IND

**Keywords:** biochemistry, chatgpt, mcqs, solving multiple choice questions, higher order cognitive skills, competency-based medical education, medical biochemistry, laboratory medicine, artificial intelligence, medical education

## Abstract

Background

Healthcare-related artificial intelligence (AI) is developing. The capacity of the system to carry out sophisticated cognitive processes, such as problem-solving, decision-making, reasoning, and perceiving, is referred to as higher cognitive thinking in AI. This kind of thinking requires more than just processing facts; it also entails comprehending and working with abstract ideas, evaluating and applying data relevant to the context, and producing new insights based on prior learning and experience. ChatGPT is an artificial intelligence-based conversational software that can engage with people to answer questions and uses natural language processing models. The platform has created a worldwide buzz and keeps setting an ongoing trend in solving many complex problems in various dimensions. Nevertheless, ChatGPT's capacity to correctly respond to queries requiring higher-level thinking in medical biochemistry has not yet been investigated. So, this research aimed to evaluate ChatGPT's aptitude for responding to higher-order questions on medical biochemistry.

Objective

In this study, our objective was to determine whether ChatGPT can address higher-order problems related to medical biochemistry.​​​​​​

Methods​​​

This cross-sectional study was done online by conversing with the current version of ChatGPT (14 March 2023, which is presently free for registered users). It was presented with 200 medical biochemistry reasoning questions that require higher-order thinking. These questions were randomly picked from the institution's question bank and classified according to the Competency-Based Medical Education (CBME) curriculum's competency modules. The responses were collected and archived for subsequent research. Two expert biochemistry academicians examined the replies on a zero to five scale. The score's accuracy was determined by a one-sample Wilcoxon signed rank test using hypothetical values.

Result

The AI software answered 200 questions requiring higher-order thinking with a median score of 4.0 (Q1=3.50, Q3=4.50). Using a single sample Wilcoxon signed rank test, the result was less than the hypothetical maximum of five (p=0.001) and comparable to four (p=0.16). There was no difference in the replies to questions from different CBME modules in medical biochemistry (Kruskal-Wallis p=0.39). The inter-rater reliability of the scores scored by two biochemistry faculty members was outstanding (ICC=0.926 (95% CI: 0.814-0.971); F=19; p=0.001)​​​​​​

Conclusion

The results of this research indicate that ChatGPT has the potential to be a successful tool for answering questions requiring higher-order thinking in medical biochemistry, with a median score of four out of five. However, continuous training and development with data of recent advances are essential to improve performance and make it functional for the ever-growing field of academic medical usage.

## Introduction

Artificial intelligence (AI) has various definitions. Still, none of them can likely adequately explain this complicated phenomenon, which is currently the focus of numerous research efforts across many scientific fields in addition to the realm of information technologies. Machines with artificial intelligence can recognize a problem and develop a workable solution using methods similar to human cognitive and psychological processes. The system learns how to accurately forecast a variable's value, classify a set of data, or carry out other challenging tasks using artificial intelligence techniques [1,2].

In recent years, numerous initiatives have been taken to use artificial intelligence and machine learning in molecular medicine, biochemistry, and cell biology [3,4]. Many machine learning models that can identify and categorize cell and tissue damage and anticipate various biochemical processes and physiological mechanisms have been developed due to the demand for automation in these areas. It is anticipated that it will take a while to incorporate most of these models into current research and therapeutic procedures. Yet, a significant number of scientists think artificial intelligence has a promising future when it comes to toxicology-based methodological approaches [5,6].

AI systems' capacity for advanced cognitive functions, including problem-solving, decision-making, reasoning, and perceiving, is called higher cognitive thinking. This thinking requires more than just processing facts; it also entails comprehending and working with abstract ideas, interpreting and applying relevant information, and producing new insights based on prior learning and experience [7]. However, it still has certain limits since, in certain studies, it has been shown not to reason creatively, comprehend emotions, or exercise moral judgment [8].

In the fields of biochemistry and laboratory medicine, AI's capacity to solve higher-order reasoning-type issues depends on the difficulty of the questions being asked and the quality of the training data the AI system has been presented with. AI systems can instantly and accurately respond to simple or uncomplicated inquiries [9]. For instance, a chatbot with a basic understanding of biochemistry may respond to inquiries about metabolic pathways, normal values of common diagnostic laboratory parameters, some traditional biomarkers of various diseases, etc. Yet, AI systems could not be as efficient as human specialists regarding more complicated queries requiring a thorough grasp of medicine and medical expertise. For instance, topics that call for inference, rational thought, and interpretation can be beyond the present capacity of systems constructed using artificial intelligence. [10].

One such AI-based conversational tool, ChatGPT, is being tested for biomedical writing and can produce replies that resemble human answers [11]. For research purposes, ChatGPT is now available for free. Recent studies by medical professionals in India showed ChatGPT to be a reliable tool for solving higher-order problems in pathology and answering first and second-order questions in microbiology using the then-recent versions of ChatGPT [12, 13]. In this study, we wanted to see how well the current version of ChatGPT handled higher-order reasoning questions in medical biochemistry.

## Materials and methods

Study setting and ethical consideration

This cross-sectional study was conducted at the biochemistry department of Dr. B.C. Roy Multi-Speciality Medical Research Centre, Indian Institute of Technology Kharagpur, over the second and third week of March 2023. The information used in this research was gathered using the recent version of Chat GPT (version March 14, 2023), presently an open-source free online tool for registered users. For data collection, we used laptops with institutional Wi-Fi broadband internet connection. There are no human research subjects in this study. As a result, the research is exempted from institutional ethical review under current norms.

Questions

As per the competencies of biochemistry in the Competency-Based Medical Education (CBME) curriculum, as designed by the National Medical Commission for Indian Medical Graduates, we divided the entire curriculum into 11 sections. This topic-wise competency mapping is shown in Table 1. 

**Table 1:** Medical biochemistry competencies as per the CBME curriculum CBME - Competency-Based Medical Education

Index	CBME competencies
BI 1	Basic biochemistry
BI 2	Enzymes
BI 3	Chemistry and metabolism of carbohydrates
BI 4	Chemistry and metabolism of lipids
BI 5	Chemistry and metabolism of proteins
BI 6	Metabolism and homeostasis
BI 7	Molecular biology
BI 8	Nutrition
BI 9	Extracellular matrix
BI 10	Oncogenesis and immunity
BI 11	Biochemical laboratory tests

Using the department's question bank, which is a compilation of first and second semester questions from various medical universities across India, we picked a total of 200 questions at random, covering the entire curriculum. Among them, 100 questions were reasoning or justification type of questions, which are typically set in first professional Bachelor of Medicine and Bachelor of Surgery (MBBS) examinations without any alternative options, and these are considered to be of higher order and an in-depth understanding of the topic is necessary to provide a solution. Instead of merely memorizing information, it focuses on fundamental ideas and principles. For instance, asking someone to apply a concept to a new circumstance requires analysis and synthesis of information as opposed in asking them to recollect a structure or description [14]. We also included 100 application-based multiple choice type questions (MCQs) from our question bank, which also required higher-order reasoning instead of simple memorization of facts. Two faculty members from the biochemistry department who have more than eight years of teaching and research experience examined the questions' face and content validity. The questions' pre-defined answer keys were included to make the evaluation more impartial.

Data collection

The process of gathering information occurred between March 14 and March 16, 2023. The questions were utilized to initiate the dialogue with ChatGPT. The response to reasoning type of questions given by the software was transcribed onto a notepad and stored on the computer to be examined later. Regarding the MCQs, we asked Chat GPT to provide the reasoning for choosing the answers. The first response was taken as final, and the option of "regenerate response" was not used. Thereafter the collected notes were printed out for evaluation. Scoring was done by two assessors on a scale of zero to five, with zero being incorrect and five being fully correct, based on a pre-selected answer key.

Statistical analysis

**Figure 1:**
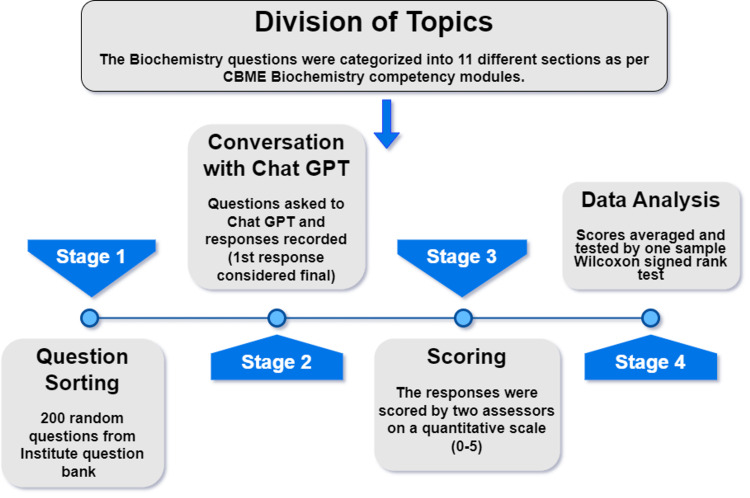
Brief outline of the study design CBME - Competency-Based Medical Education

The data were expressed as number, mean, median, standard deviation, and first and third quartiles using descriptive statistical tests. The Kolmogorov-Smirnov test was performed with the dataset, which showed the distribution was not normal. We conducted a one-sample Wilcoxon signed rank test using hypothetical anticipated values to assess the response's correctness. The intraclass correlation coefficient was used to evaluate the score between the two raters (ICC). For all of the statistical analyses, we utilized SPSS version 21 (IBM Inc., Armonk, New York). A p-value of less than 0.05 was regarded as statistically significant. A brief outline of the study design has been highlighted in Figure 1.

## Results

**Figure 2:**
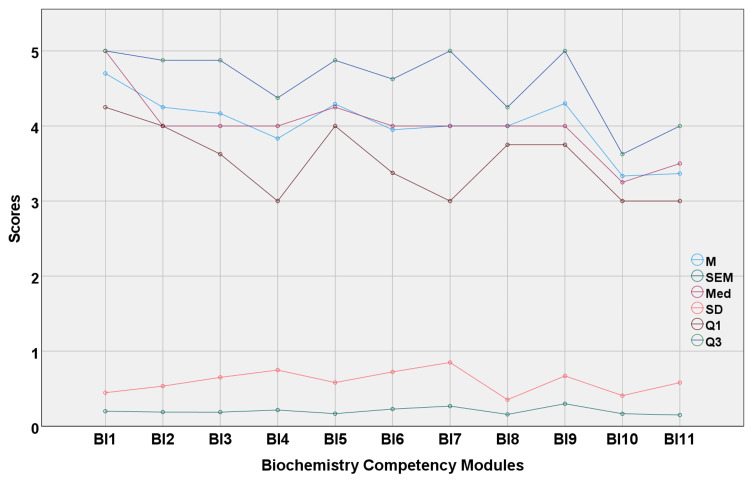
Multiple line diagram showing comparative statistical analysis of the score of various modules BI 1 to BI 11 represent the entire biochemistry curriculum as per CBME which was shown in Table 1. BI - biochemistry competency modules, M - mean, SEM - standard error of the mean, Med - median, SD - standard deviation, Q1 - first quartile, Q3 - third quartile, CBME - Competency-Based Medical Education

The AI software answered a total of 200 questions requiring higher-order thinking with a mean score of 3.98 (± 0.70) and a median score of 4.0. (Q1-3.50, Q3-4.50). The overall and system-wise scores of the responses are shown in Table 2 and graphically represented in Figure 2. 

**Table 2:** Statistical analysis of scores of various competency modules as per the competency-based medical education curriculum in biochemistry BI - biochemistry competency module category, SD - standard deviation, SEM - standard error of the mean, Q1 - first quartile, Q3 - third quartile

Topics	Mean	SD	SEM	Median	Q1	Q3	One sample Wilcoxon signed rank test	
							p-value (hypothetical median 4)	p-value (hypothetical median 5)
BI 1 (n=10)	4.70	0.45	0.20	5.00	4.25	5.00	0.059	0.180
BI 2 (n=16)	4.25	0.53	0.19	4.00	4.00	4.88	0.194	0.024
BI 3 (n=24)	4.17	0.65	0.19	4.00	3.63	4.88	0.351	0.007
BI 4 (n=24)	3.83	0.75	0.22	4.00	3.00	4.38	0.420	0.005
BI 5 (n=24)	4.29	0.58	0.17	4.25	4.00	4.88	0.140	0.006
BI 6 (n=20)	3.95	0.72	0.23	4.00	3.38	4.63	0.862	0.011
BI 7 (n=20)	4.00	0.85	0.27	4.00	3.00	5.00	1.000	0.017
BI 8 (n=10)	4.00	0.35	0.16	4.00	3.75	4.25	1.000	0.039
BI 9 (n=10)	4.30	0.67	0.30	4.00	3.75	5.00	0.276	0.102
BI 10 (n=12)	3.33	0.17	0.17	3.25	3.00	3.63	0.038	0.026
BI 11 (n=30)	3.37	0.15	0.15	3.5	3.00	4.00	0.004	< 0.001
Total (n=200)	3.98	0.70	0.07	4.0	3.50	4.50	0.16	0.001

Using a single sample Wilcoxon signed rank test, the result was less than the hypothetical maximum of five (p=0.001) and comparable to four (p=0.16). The median score of BI 1 was near to five, and the median score of modules BI 10 and BI 11 was below four (3.25 and 3.5, respectively). There was no difference in the replies to questions from different CBME modules in medical biochemistry (Kruskal-Wallis p=0.39). The p-values from the post hoc test were not presented because there was no discernible difference between the two groups. The inter-rater reliability of the scores scored by two biochemistry faculty members was outstanding. The ICC was 0.926 with a 95% confidence interval of 0.814 to 0.971 (F=19; p<0.001). A few examples of Chat GPT responses are shown in the appendix.

## Discussion

We tested ChatGPT's ability to solve complex biochemistry questions by giving it 200 random questions. Based on our findings, ChatGPT's responses scored at least four out of five marks on all the modules except the last two modules, where the score was lower. Numerous past investigations have evaluated the potential of ChatGPT for medical education purposes. The observation is similar to a recent study conducted by Sinha et al. demonstrating the utility of ChatGPT for solving higher-order problems in pathology [12]. The study conducted by Das et al. had a similar outcome where ChatGPT was shown to answer first and second-order questions of microbiology with 80% accuracy, thereby corroborating with our study [13]. According to Gilson et al., ChatGPT is able to provide answers to medical queries through natural language processing, equivalent to that of a third-year medical student in the United States. In addition, they noted that ChatGPT has the ability to provide reasoning and informative context in most of its responses, which is due to its dialogic nature when answering questions [15]. For this research, we utilized the kind of reasoning-based questions that are frequently asked of first-year medical students attending medical colleges in India. The findings from Gilson's study align with the results of our study. Additionally, Kung et al. conducted a study that discovered that ChatGPT could successfully complete the United States Medical Licensing Examination without any human assistance. Moreover, ChatGPT demonstrated clear, logical thinking and provided accurate clinical insights in its responses [16].

On the other hand, Huh's study discovered that ChatGPT's performance in parasitology is still lacking compared to that of a Korean student [17]. The study by Juhi et al. also shows that ChatGPT is only partially reliable in predicting and explaining drug-drug interactions in pharmacology [18]. Furthermore, we have noticed that the scores for the last two modules were lower than those for the others. This could be because these modules, namely BI 10 (focused on cancer and immunology) and BI 11 (centered on biochemical laboratory tests), involve newer developments, such as ongoing research on cancer and advancements in clinical biochemistry that may not have been included in the dataset used to train ChatGPT.

We recommend that medical schools and colleges should not impose limitations on the utilization of AI. Instead, the students should be equipped with the necessary skills to use them optimally. This is of utmost importance that when a student is trying to solve MCQs, they can readily get the answer from ChatGPT with proper reasoning. Simply pasting an MCQ the same into an online search engine may not give any useful response. It is imperative to ensure that future AI systems are designed, developed, and validated meticulously to provide medical students with reliable and precise information. As AI continues to advance, there is a need for further development in health-related information to increase its potential to be incorporated into education and healthcare systems [19]. It is a fact that Chat GPT relies on a dataset of information that only goes up to 2021, which could be deemed outdated since its output may not incorporate the latest advancements, as observed in the responses in some of the modules in our study. It is crucial to monitor and update AI systems regularly to guarantee that they stay pertinent and current with the constant developments in biochemistry and molecular biology, which is an integral part of evidence-based modern medicine and research.

Limitations

There are various limitations to this study. Firstly, we employed a scoring approach that ranged from zero to five. Despite preparing the answer keys beforehand, there may still have been a subjective bias in the assessment that was beyond our control. The questions used in our study were sourced from our question bank, and other institutions may have different questions. Therefore, future studies may need to be conducted multicentrically for a more generalizable outcome. Furthermore, even a slight modification to a question may result in a different response from ChatGPT, so this should be considered in future investigations.

## Conclusions

It can be concluded that ChatGPT helps in seeking answers for higher-order reasoning questions in medical biochemistry. The study showed a median score of four out of five in solving the questions with better performance in explaining traditional concepts rather than justifying recent advances due to its limitation in training databases in the constantly evolving medical research in cancer biochemistry, immunology, and clinical investigations. It can be an excellent tool for solving multiple-choice questions and getting the proper reasoning behind the solution. Although such cognitive ability in AI can benefit students and academicians seeking quick and functional responses to their inquiries, considering the evolution of AI programs worldwide, testing their capabilities in future studies in various medical disciplines is imperative.

## Figures and Tables

**Figure 3:**
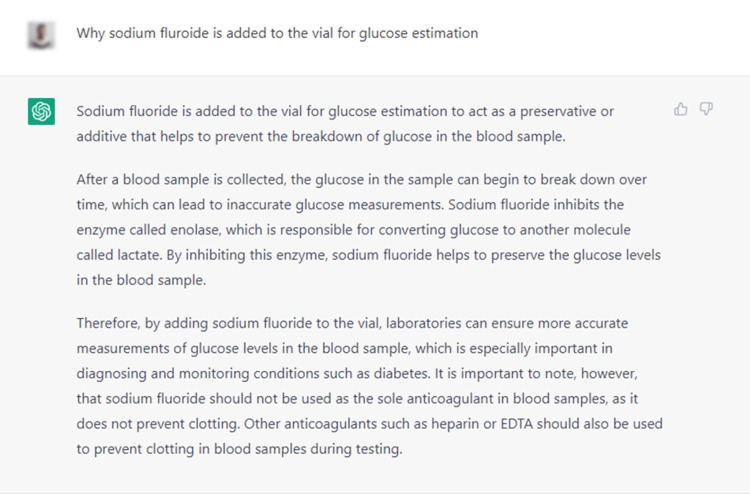
Partial screenshot of a conversation with ChatGPT with higher-order reasoning questions with descriptive answers EDTA - ethylenediaminetetraacetic acid The registered user profile picture has been blurred to prevent identification and facilitate blinding during peer review

**Figure 4:**
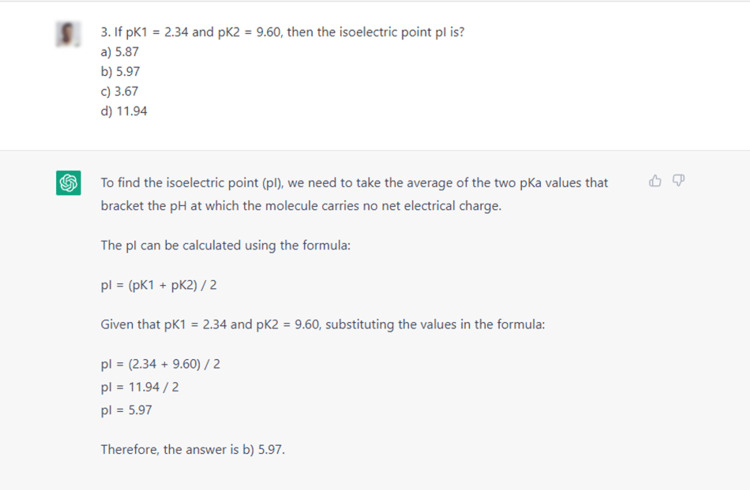
Partial screenshot of a conversation with ChatGPT with multiple choice type questions requiring higher-order reasoning pH = potential of hydrogen, pI = isoelectric point, pKa = dissociation constant, pK1 = dissociation constant 1, pK2 = dissociation constant 2 The registered user profile picture has been blurred to prevent identification and facilitate blinding during peer review

## References

[1] Dimitriadis I, Zaninovic N, Badiola AC, Bormann CL (2022). Artificial intelligence in the embryology laboratory: a review. Reprod Biomed Online.

[2] Crigger E, Reinbold K, Hanson C, Kao A, Blake K, Irons M (2022). Trustworthy augmented intelligence in health care. J Med Syst.

[3] Wang JX, Wang Y (2020). Towards machine learning in molecular biology. Math Biosci Eng.

[4] Hudson IL (2021). Data integration using advances in machine learning in drug discovery and molecular biology. Methods Mol Biol.

[5] Singh AV, Romeo A, Scott K (2021). Emerging technologies for in vitro inhalation toxicology. Adv Healthc Mater.

[6] Davidovic LM, Laketic D, Cumic J, Jordanova E, Pantic I (2021). Application of artificial intelligence for detection of chemico-biological interactions associated with oxidative stress and DNA damage. Chem Biol Interact.

[7] Zhao J, Wu M, Zhou L, Wang X, Jia J (2022). Cognitive psychology-based artificial intelligence review. Front Neurosci.

[8] Jiang L, Wu Z, Xu X, Zhan Y, Jin X, Wang L, Qiu Y (2021). Opportunities and challenges of artificial intelligence in the medical field: current application, emerging problems, and problem-solving strategies. J Int Med Res.

[9] Sharma M, Savage C, Nair M, Larsson I, Svedberg P, Nygren JM (2022). Artificial intelligence applications in health care practice: scoping review. J Med Internet Res.

[10] Korteling JE, van de Boer-Visschedijk GC, Blankendaal RA, Boonekamp RC, Eikelboom AR (2021). Human- versus artificial intelligence. Front Artif Intell.

[11] van Dis EA, Bollen J, Zuidema W, van Rooij R, Bockting CL (2023). ChatGPT: five priorities for research. Nature.

[12] Sinha RK, Deb Roy A, Kumar N, Mondal H (2023). Applicability of ChatGPT in assisting to solve higher order problems in pathology. Cureus.

[13] Das D, Kumar N, Longjam L (2023). Assessing the capability of ChatGPT in answering first- and second-order knowledge questions on microbiology as per competency-based medical education curriculum. Cureus.

[14] Lemons PP, Lemons JD (2013). Questions for assessing higher-order cognitive skills: it's not just Bloom's. CBE Life Sci Educ.

[15] Gilson A, Safranek CW, Huang T, Socrates V, Chi L, Taylor RA, Chartash D (2023). How does ChatGPT perform on the United States Medical Licensing Examination? The implications of large language models for medical education and knowledge assessment. JMIR Med Educ.

[16] Kung TH, Cheatham M, Medenilla A (2023). Performance of ChatGPT on USMLE: potential for AI-assisted medical education using large language models. PLOS Digit Health.

[17] Huh S (2023). Are ChatGPT’s knowledge and interpretation ability comparable to those of medical students in Korea for taking a parasitology examination?: a descriptive study. J Educ Eval Health Prof.

[18] Juhi A, Pipil N, Santra S (2023). The capability of ChatGPT in predicting and explaining common drug-drug interactions. Cureus.

[19] Xu L, Sanders L, Li K, Chow JC (2021). Chatbot for health care and oncology applications using artificial intelligence and machine learning: systematic review. JMIR Cancer.

